# Splicing Anomalies in Myeloproliferative Neoplasms: Paving the Way for New Therapeutic Venues

**DOI:** 10.3390/cancers12082216

**Published:** 2020-08-07

**Authors:** Marie Hautin, Clélia Mornet, Aurélie Chauveau, Delphine G. Bernard, Laurent Corcos, Eric Lippert

**Affiliations:** 1 Inserm, Univ Brest, EFS, UMR 1078, GGB, F-29200 Brest, France; marie.HautinRopert@univ-brest.fr (M.H.); aurelie.chauveau@chu-brest.fr (A.C.); delphine.bernard@univ-brest.fr (D.G.B.); laurent.corcos@univ-brest.fr (L.C.); 2 Laboratoire d’Hématologie, CHU de Brest, F-29200 Brest, France; clelia.mornet@gmail.com

**Keywords:** splicing, myeloproliferative neoplasm, epigenetic

## Abstract

Since the discovery of spliceosome mutations in myeloid malignancies, abnormal pre-mRNA splicing, which has been well studied in various cancers, has attracted novel interest in hematology. However, despite the common occurrence of spliceosome mutations in myelo-proliferative neoplasms (MPN), not much is known regarding the characterization and mechanisms of splicing anomalies in MPN. In this article, we review the current scientific literature regarding “splicing and myeloproliferative neoplasms”. We first analyse the clinical series reporting spliceosome mutations in MPN and their clinical correlates. We then present the current knowledge about molecular mechanisms by which these mutations participate in the pathogenesis of MPN or other myeloid malignancies. Beside spliceosome mutations, splicing anomalies have been described in myeloproliferative neoplasms, as well as in acute myeloid leukemias, a dreadful complication of these chronic diseases. Based on splicing anomalies reported in chronic myelogenous leukemia as well as in acute leukemia, and the mechanisms presiding splicing deregulation, we propose that abnormal splicing plays a major role in the evolution of myeloproliferative neoplasms and may be the target of specific therapeutic strategies.

## 1. Introduction

Myeloproliferative neoplasms (MPNs) are clonal myeloid malignancies characterized by excessive proliferation of myeloid progenitors due to increased response to hematopoietic growth factors. They are typically divided in two main categories: (a) chronic myelogenous leukemia (CML), characterized by the fusion of *BCR* and *ABL1* genes, leading to the generation of a chimeric, constitutively active tyrosine-kinase, BCR-ABL1 [1,2]. Most often, the gene fusion is the result of a 9;22 translocation, generating the so-called “Philadelphia chromosome” (derivative chromosome 22) [2,3]. The constitutively active kinase BCR-ABL1 drives the uncontrolled proliferation of myeloid cells, particularly of the granulocytic lineage [4]. (b) By contrast, other MPN are named “Philadelphia negative”, because even though they share pathophysiological characteristics with CML (bone marrow hypercellularity, cytokine independence) [5], they do not display a Philadelphia chromosome (hereafter referred to as “Philadelphia-negative MPN”). Three main diseases can be distinguished depending on the primarily affected myeloid lineage: erythrocytes are increased in polycythemia vera (PV), platelets in essential thrombocythemia (ET), and both can evolve into myelofibrosis (MF) (post-PV or post-ET MF), which may also occur de novo (prefibrotic or overt primary myelofibrosis, PMF) [6,7]. In addition to the risk of evolution of ET to PV and ET or PV to MF, all MPN may evolve towards acute myeloblastic leukemia (AML) [6], a condition with a very poor prognosis. In untreated CML patients, blastic transformation is constant within a few years [8]. However, the vast majority of patients respond to tyrosine-kinase inhibitors (TKIs) and transformation has become the exception. By contrast, not all Philadelphia-negative MPN will transform and generally, after a longer period.

The molecular mechanisms involved in myeloid proliferation mainly affect growth factor signaling pathways. In CML, it is the constitutive oligomerization of the tyrosine kinase ABL1 which, due to its fusion to the dimerization domain-containing BCR, triggers hyperproliferation [9]. In Philadelphia-negative MPN, several mutations have been described, all of which mainly converge onto the JAK2/STAT5 pathway [6]. The first of these mutations was discovered in 2005, in the exon 14 of the *JAK2* gene (*JAK2*^V617F^) [10,11,12,13]. This tyrosine kinase is involved in transducing signals from erythropoietin (EPO), thrombopoietin (TPO) and granulocyte-colony stimulating factor (G-CSF) receptors [5,6]. The point mutation results in relieving the negative control of the pseudo-kinase domain on the catalytic domain, thus leading to cytokine hypersensitivity or even independence [10,12,13], a hallmark of MPN. The *JAK2*^V617F^ mutation can be found in all three types of Philadelphia-negative MPN, accounting for the vast majority of PV mutations (while a small proportion (1–2%) is due to alternative mutations in the exon 12 of *JAK2*), and 50–60% in ET or PMF [5,6]. In ET or PMF, mutations can also be found in the gene coding for the thrombopoietin receptor itself (MPL, 5–10%) [5,6,14,15,16], or in the gene coding for the ER resident quality control protein CALR (25–40%) [5,17,18]. Mutations in these genes result in constitutive signaling of the TPO/MPL axis [19,20,21,22,23], thus promoting megakaryocytic proliferation and differentiation. These mutations, shown to be sufficient for the development of the myeloproliferative phenotype, especially in murine models, are generally referred to as “driver mutations” [6]. A proportion of ET and PMF which do not display these driver mutations are called “triple negative”.

In addition to driver mutations, recent data mainly obtained by Next Generation Sequencing, indicate that “additional mutations” are recurrently found, particularly in genes involved in epigenetic and splicing regulation [6]. These additional mutations are commonly found in all types of myeloid neoplasias. Their presence and even more their accumulation is frequently associated to a more aggressive course [6,24]. In Philadelphia-negative MPN, this is particularly true for mutations affecting *ASXL1*, *SRSF2*, *EZH2* or *IDH1/2* [6,25]. Mutations affecting epigenetic regulators may alter DNA methylation (*DNMT3A*), hydroxymethylation (*TET2*, *IDH1/2*) or histone post-translational modifications (*ASXL1*, *EZH2*, *IDH1/2*) [5]. In murine models, reproduction of mutations in these genes generally results in increased hematopoietic stem cell (HSC) self-renewal, thus contributing to the leukemic phenotype [26]. However, by themselves, these mutations have a founding effect, but not sufficient to promote leukemogenesis. This is further supported by the fact that epigenetic mutations are the most frequently encountered in clonal hematopoiesis of indeterminate potential (CHIP) [27,28].

**Figure 1:**
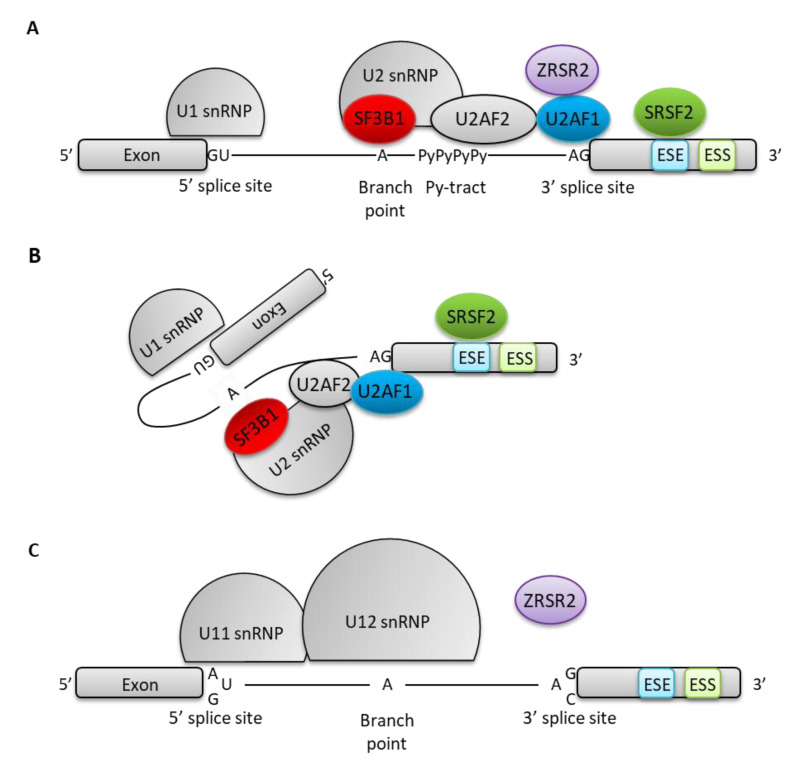
Schematic representation of spliceosome complexes. (**A**) Representation of U1 snRNP, U2 snRNP and auxiliary factors fixation on pre-mRNA (major spliceosome). U1 snRNP recognizes the 5′-splice site. U2snRNP recognizes the 3′-splice site at conserved sequence positions: the branch-point, the poly-pyrimidine-tract (Py-tract), and the AG dinucleotide thanks to SF3B1 in SF3B complex, U2AF2 and U2AF1 respectively. SRSF2 binds to exonic splicing enhancer (ESE) to favor recognition and fixation of U1 and U2 snRNP. ZRSR2 promotes major spliceosome assembly. (**B**) U1 snRNP and U2 snRNP joining allows the 5′-splice site to interact with the branch point. (**C**) U11 and U12 snRNP belong to the minor spliceosome that is involved in U12-intron splicing. U12-introns are delimited by sequences at 5′ (GU or AU) and 3′ (AG or AC) splice sites different from those present in U2-introns. ZRSR2 promotes minor spliceosome assembly.

Additional mutations also affect genes involved in splicing regulation. These mutations are commonly found in myelodysplastic syndromes (MDS) and in some AML where they define specific entities and participate in the ontogeny of secondary AML [29,30]. Splicing is the mechanism leading from pre-messenger RNA to mature mRNA by removal of intronic regions [31]. It relies on correct definition of exons, guided by specific and conserved sequences known as the 5′donor and 3′acceptor consensus sequences (typically GU and AG) that can be recognized by specific small nuclear ribonucleoprotein (snRNP) complexes, U1 and U2 respectively, in association with auxiliary factors forming a large ribonucleoparticle complex called spliceosome [31]. The U2 subunit binds a conserved adenosine, called “branch point”, localized 5′ from the acceptor site and its upstream polypyrimidine-tract (Figure 1A) [31]. This binding relies on the recognition of the acceptor site by the U2AF1 protein, and the polypyrimidine-tract by U2AF2, in the U2AF complex [31]. This association constitutes the E (Early) complex of the spliceosome [31]. In the next step, U2 is recruited to the branch point via its component SF3B1 and eventually joins U1snRNP, thus allowing the donor site to link the branch point (Figure 1B) through a non-canonical 2′–5′ transesterification, forming a lariat which will be eliminated after a regular 5′–3′ transesterification between the donor and the acceptor sites [31]. For some introns, an alternative complex (minor spliceosome) uses U11 and U12 instead of U1 and U2 (Figure 1C) [32,33]. This minor spliceosome relies on the ZRSR2 protein for correct splicing [34].

**Figure 2:**
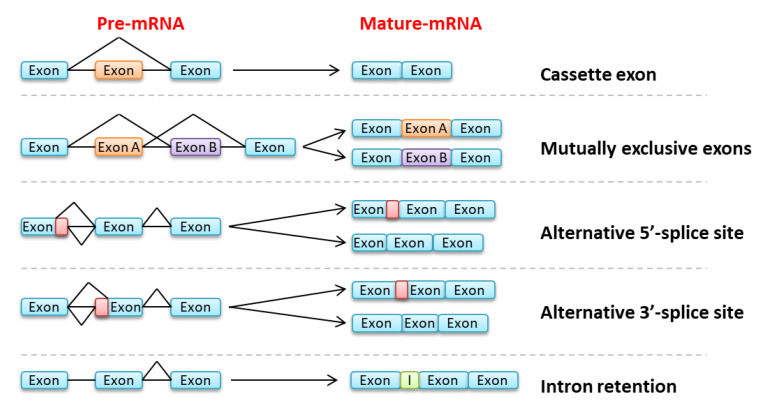
Examples of splicing events. From the top: cassette exon, mutually exclusive exons, alternative 5′ and 3′-splice sites, and intron retention.

This mechanism of intron removal can be modulated to generate multiple mature transcripts from the same pre-mRNA, thus increasing dramatically protein diversity [31]. It is estimated that around 80,000 proteins [35] can be generated from around 20,000 genes in humans [36]. Alternative splicing can be due to mutually exclusive exons, cassette exons, alternative 5′ or 3′ splice sites or intron retention (Figure 2) [31]. It may result in an alternative protein sequence with functions that can be different, and sometimes opposite to those translated from the canonical transcript. Alternatively, the transcript may be targeted for nonsense-mediated mRNA decay (NMD) if a STOP codon is introduced, thus resulting in a downregulation of the protein expression [37]. A strict regulation is necessary to control different splicing patterns characteristic of cell type and maturation stage [38]. This has been particularly well demonstrated in the hematopoietic system. Splicing regulation can be effected by various factors including transcription rate or binding of splicing regulators which mainly belong to two protein families: SR proteins (comprising serine- and arginine-rich domains) mostly involved in exon inclusion by binding enhancer sequences (intronic or exonic splicing enhancers, ISE or ESE) and heterogeneous nuclear ribonucleoproteins (hnRNP), favoring exon skipping through binding intronic and exonic splicing silencers (ISS or ESS) [39].

Splicing deregulation has been thoroughly described in cancers, especially in hematopoietic malignancies. As mentioned above, this may be due to mutations affecting spliceosome proteins, but also to mutations in cis, involving 5′/3′ splice sites, ISE, ISS, ESE or ESS. Moreover, even in the absence of specific mutations, splicing programs have been shown to be deregulated in cancers, possibly reversing them to immature cell patterns [40]. The causes of these deregulations are not entirely elucidated, but at least partly rely on changes in epigenetic programs [41]. More specifically, in myeloid disorders, splicing deregulation is a major feature of MDS, mainly owing to splicing factor mutations [42], but has also been well characterized in AML or CML, where splicing programs can be modified in the absence of spliceosome mutations [43,44]. Little is known about splicing deregulations in Philadelphia-negative MPN, but accumulation of splicing anomalies during CML evolution and splicing programs characteristic of AML suggest that splicing abnormality probably plays a role in MPN, particularly in explaining adverse evolutions. This article is aimed at reviewing current knowledge about splicing abnormalities in Philadelphia-negative MPN. When necessary for pathophysiological approaches, examples in MDS, CML and/or AML will also be described. A Pubmed database search for articles containing the following keywords: “Splicing and myeloproliferative” retrieved 146 articles which were then screened and classified as “clinical series” and “pathophysiology”. For the clinical series approach, only articles reporting series of MPN patients with spliceosome mutations were analyzed (*n* = 26). For both categories, additional articles cited in the references and containing relevant information were also included. In a first part, we will discuss the clinical consequences of splicing factor mutations in Philadelphia-negative MPN. We will then rapidly present the current knowledge on pathophysiological consequences of these mutations and their role in leukemogenesis. Next, splicing abnormalities in myeloid malignancies in the absence of spliceosome mutations will be presented, with a focus on Philadelphia-negative MPN. Elements of molecular explanations for these anomalies will be proposed and venues for therapeutic developments will be discussed.

## 2. Clinical Correlates of Spliceosome Mutations in MPN

Additional mutations in myeloid malignancies have been described in many publications, owing mainly to the availability of next generation sequencing (NGS) techniques. In MPN, these publications constantly report the predominance of mutations affecting epigenetic regulators, including *ASXL1* (22–to 36%, 4–11% and 3–12% in PMF, ET and PV), *TET2* (18–22%, 11–16% and 15–22%), and *DNMT3A* (5–9%, 6% and 2–4%) [45,46,47]. Mutations in RNA splicing factors *SF3B1*, *U2AF1*, *SRSF2*, *ZRSR2* and *PRPF8* represent the second most important category in MPN. Data of the 26 selected studies have been grouped in order to calculate an overall frequency (Table 1). Mutations are more frequent in PMF than in ET and PV. In PMF, *SRSF2* and *U2AF1* mutations were the most frequent: 12.2% and 13.8% respectively, followed by *SF3B1* (7.7%) and *ZRSR2* (5.8%) mutations, whereas *PRPF8* (2.1%) mutations were rarer. Since the recent introduction of the pre-MF entity, few large series are available. However, they confirm that *SRSF2* mutations are found in this early stage of the pathology at a frequency of 8.5% (Table 1).

Splicing factor mutations were less frequent in PV and ET than in PMF (*p* < 0.0001 for all mutations) and their repartition also seemed to differ. Indeed, *SF3B1* mutations were found in 2.8% and 1.4% (*p* = 0.011 for ET vs. PV), *SRSF2* mutations in 1.5% and 1.8% (*p* = 0.4 for ET vs. PV), *U2AF1* mutations in 1.1% and 0.3% (*p* = 0.03 for ET vs. PV), *ZRSR2* in 0.7% and 1.3% (*p* = 0.18 for ET vs. PV), of ET and PV respectively. *PRPF8* mutations have not been screened for in large cohorts but were found in only one of 35 ET patients; and none of eight PV (Table 1). When PV and ET progressed to MF, frequencies increased with 2.6% of patients with secondary MF (SMF) harbouring *SRSF2* mutations (*p* = 0.27 and 0.08 compared to PV and ET respectively), 6.9% *SF3B1* mutations (*p* < 0.0001 and *p* = 0.012), 4.9% *U2AF1* mutations (*p* < 0.0001 and *p* = 0.0007), 6.9% *ZRSR2* mutations (*p* = 0.005 and *p* < 0.0001) and, even though data come from a small cohort, 1.6% *PRPF8* mutations (Table 1).

MPN may also transform to AML. Mutations in RNA splicing factors were found in post-MPN AML with a frequency similar to PMF: *SRSF2* mutations were observed in 17% of post-MPN AML (post-ET, -PV and -PMF AML, combined, *p* = 0.09 for PMF vs. AML), 8.1% for *SF3B1* (*p* = 0.87 for PMF vs. AML), 9.5% for *U2AF1* (*p* = 0.15 for PMF vs. AML), 1.1% for *ZRSR2* and 5.3% for *PRPF8* mutations (Table 1). The frequent occurrence of spliceosome mutations in MF or in post-MPN AML suggests that these mutations are associated with advanced phases and may transform cells further.

Regarding mutation co-occurrences, all types of MPN (*JAK2*-, *CALR*- or *MPL*-mutated as well as triple negative MPN) are affected by spliceosome mutations. However, in MF and in ET, *CALR*-mutated MPN were under-represented among *SRSF2*-mutated patients (3.5 and 5.9% respectively, Table S1; versus 33% and 23% expected for *CALR*-mutated MF and ET) and only one of 17 *SRSF2*-mutated patients had a triple-negative ET (15% expected). The negative correlation between *CALR* and *SRSF2* mutations had already been highlighted by Tefferi et al. [45] in PMF, but it seems to hold for ET patients. Regarding association of “additional” mutations, the frequent co-occurrence of *SRSF2* and *ASXL1* mutations had also been described in PMF [24]. Overall, 42.4% of *SRSF2*-mutated PMF patients also had *ASXL1* mutation, similar to ET (41.2%). However, *ASXL1* mutations were even more frequent in *U2AF1*-mutated PMF (63.4%) as expected [45,48,49], but not ET patients (7.1%). *IDH1/2* mutations have also been found associated with *SRSF2* mutations, in MPN as well as in AML [24,45,50,51]. This association is confirmed in ET as well as in PMF. In contrast, *DNMT3A* mutations were not found in *SRSF2*-mutated ET (whereas they associate with other splicing mutations) and are less frequently found in *SRSF2*-mutated PMF patients (4.7%) than in patients with other splicing mutations (11.7% and 8.5% for *SF3B1-* and *U2AF1*-mutated patients). Given the very small numbers, it is difficult to draw definitive conclusions regarding the co-occurrence of other additional mutations, however, signaling mutations seem more frequent in *SRSF2*- and *U2AF1*-mutated MF, but not ET and the absence of other additional mutation is less frequent in *SRSF2*-mutated ET (24% vs. 50–60%), but not MF.

Regarding myelodysplastic/myeloproliferative neoplasms, MDS/MPN with ring sideroblasts and thrombocytosis (formerly refractory anemia with ring sideroblasts associated with marked thrombocytosis [RARS-T]) was frequently associated with *SF3B1* mutations [65,66,67]. Finally, for comparison, in the analysed articles, around 33% of chronic myelomonocytic leukemia (CMML) patients harbored *SRSF2* mutations [42,68]; 8% *U2AF1* mutations, 9% *ZRSR2* mutations [42] and 5%, *SF3B1* mutations [42,65]. *SRSF2* mutations being the most frequent splice mutations in MPN, particularly in PMF, they were the only events for which clinical correlates have been extensively studied. Even so, there persists some discrepancies between the series: two studies gathering 569 patients described a significant association between *SRSF2* mutations and leukocytosis > 25G/L [24,48] in PMF, whereas it was not found in two others [45,51]. However, in the study by Vannucchi et al. [24], among two cohorts (one from Europe, the other from the Mayo Clinic), only the European cohort showed this significant association. In addition, the study by Courtier et al. [48] did not differentiate PMF from SMF. Overall, considering the 765 patients reported in these series, the correlation with high leukocyte counts seems rather weak. Similarly, two studies (grouping 578 patients) described a correlation between *SRSF2* mutations and anemia/transfusion requirement [24,45] in PMF, but this correlation was not significant in a third one [51]. However, in this study by Lasho et al. [51], the association between *SRSF2* mutations and transfusion requirement was close to being significant (*p* = 0.06). We are therefore rather led to believe that there is a correlation between *SRSF2* mutations and transfusion requirement, even though a significant association was found only in one (Mayo Clinic cohort) of the two cohorts reported by Vannucchi et al. [24]. Last, according to one study (on 483 patients), the percentage of circulating blasts was higher in *SRSF2* mutated patients [24]; whereas this correlation was not present in another study [51]. All reports agreed that there was no established association between low platelet counts and the presence of *SRSF2* mutations [45,48,51]. On the other hand, some associations were constantly found between the different studies. *SRSF2* mutations were associated with advanced age, regardless of the study (a total of 1066 patients) [24,51]. Most of the Dynamic International Prognostic Scoring System Plus (DIPSS Plus) prognostic markers were associated with the presence of *SRSF2* mutations: age older than 65 years, hemoglobin lower than 10 g/dL, leukocytes higher than 25 G/L, circulating blasts over 1%, and constitutional symptoms (the association was statistically significant in the European cohort of [24] and in [45] (*n* = 665 patients), but not in [51] and the Mayo Clinic cohort of [24] (*n* = 583 patients)); but also karyotype, platelet count and transfusion status. It is therefore not surprising to find a significant association between *SRSF2* mutations and high-risk DIPSS Plus score [24,45,51].

As expected, given the correlation with DIPSS Plus scores, a lower overall survival in patients with *SRSF2*-mutated PMF was constantly found [24,45,48,51,52]. Shorter overall survival was also described in *SRSF2*-mutated ET and PV [46,53], post-ET MF [54], as well as in post-MPN AML (regardless of previous MPN) [50,55]. *SRSF2* mutations were also associated with a lower leukemia-free survival in MPN [50], more particularly in PMF [24,45,51]. Regarding ET and PV, associations were not as obvious: the ET cohort of the Mayo Clinic (*n* = 270 patients) showed lower leukemia-free survival in mutated patients, but this association was not significant in the European cohort (*n* = 232 patients) [53] or in another cohort (*n* = 183 patients) of the Mayo Clinic [46]. Regarding MF-free survival, a significant association with *SF3B1* mutations was described in the European cohort [53], but not in the two Mayo Clinic cohorts [46,53]. Concerning PV, no conclusions can be drawn for leukemia-free survival, the two cohorts of each article showing opposite trends [46,53]. However, there appears to be no correlation between *SRSF2* mutations and MF-free survival in PV patients. The association, although described in a single cohort (*n* = 133 patients) [46], was not found in a control cohort presented in the same article, or in any of the two cohorts of [53] (a total of 619 patients).

Regarding mutations of other RNA splicing factors, as reported in MDS, *SF3B1* mutations were associated with the presence of ring sideroblasts [56,62], even leading to the proposition of a specific MDS entity [69], but in contrast to MDS, there was no positive impact of *SF3B1* mutation on overall or leukemia-free survival in PMF [48,62] or in PV [53] patients. Moreover, *SF3B1*-mutated ET patients had a lower overall and MF-free survival [46,53]. Last, *U2AF1* mutations correlated with anemia and thrombocytopenia [45,48,49,57] in PMF patients, and were thus associated with a lower overall survival. This difference was found in univariate analysis but did not hold in multivariate analysis including anemia and/or thrombocytopenia as covariables. No significant correlation exists between *U2AF1* status and overall or leukemia-free survival in ET or PV but *U2AF1*-mutated ET seem to progress more rapidly towards SMF [46,53]. In conclusion, *SF3B1*, *SRSF2*, *ZRSR2* and *U2AF1* spliceosomal mutations affect overall and leukemia-free survival in MPN [58].

## 3. Consequences of Splicing Anomalies in Myeloid Malignancies

### 3.1. Molecular Mechanisms of Spliceosome Mutations

Spliceosome mutations have initially been identified after exome sequencing in MDS [42,70,71]. This unexpected finding highlights the role of abnormal splicing in the pathogenesis of myeloid disorders. These mutations have since been described in MPN as well as in non-myeloid cancers (CLL, solid cancers) [72]. They mostly generate aberrant transcripts or change the equilibrium between normally occurring isoforms. How these alterations translate into myeloid malignancies is not completely understood, but various mechanisms have been proposed for each spliceosome gene mutation, mainly in the context of MDS [73].

In MPN, *SRSF2* is the most frequently mutated spliceosome-interacting component. This member of the SR-protein family is characterized by the presence of a RNA recognition motif (RRM) involved in binding ESE [74] and an arginine/serine-rich (RS) domain that plays a role in protein interactions [74]. SRSF2 also facilitates U2AF complex recruitment at the 3′-splice site and U1 snRNP at the 5′-splice site [74], thus promoting exon recognition and inclusion. Mutations in *SRSF2* are mostly found in the sequence between the RRM and RS domains, with a hotspot at proline 95 [75,76]. Rare mutations affect other residues than P95, with similar consequences for those affecting the 20 amino-acids surrounding it [77]. These mutations alter the affinity of SRSF2 for specific sequences in ESE, leading to a deregulation of exon exclusion/inclusion [77,78,79,80,81]. Exons containing C-rich sequences in their ESE are more frequently included in *SRSF2*-mutated cells, since the SRSF2 mutant proteins bind to RNA sites containing the consensus UCCAG with greater affinity than wild-type SRSF2, while exons containing G-rich sequences are more frequently excluded [77,78,80,81]. *SRSF2* mutations affect global splicing regulation since many actors of splicing, including SR and hnRNP family proteins are themselves the targets of mutant SRSF2-induced missplicing [78]. In addition, *SRSF2* mutations impact on epigenetic regulation by promoting the inclusion of a “poison cassette exon” in *EZH2* that introduces a premature STOP codon, thus targeting the transcript for NMD [81]. The ensuing decrease in *EZH2* expression, a component of the Polycomb Repressive complex 2 (PRC2) histone modifying complex, favors the development of MDS [82], initiation of MPN and development of MF, as observed in the case of *EZH2* mutations [83]. However, not all MDS patients with *SRSF2*^P95H^ mutation show significant inclusion of this poison exon, suggesting that other mechanisms are at play [78]. Mice in which the *SRSF2*^P95H^ has been introduced in hematopoietic cells by knock-in strategies display various MDS characteristics including macrocytic anemia, block in erythroid differentiation, multi-lineage dysplasia, but also features reminiscent of MPN such as increased LT-HSC, increased myeloid cells in peripheral blood, monocytosis or increased splenic erythropoiesis [79,81,84].

U2AF1 is an U2 auxiliary factor protein involved in recognizing the 3′ boundary of introns at the AG splice site acceptor dinucleotide [85]. Its role in determining exon boundaries is crucial for the selection of spliced exons [86]. Hotspot mutations in *U2AF1* lead to substitutions located in two highly conserved zing fingers, at S34 and Q157 [71,76] and are associated with distinct patterns of exon inclusion [77,87]: S34 mutations affect binding of U2AF1 to the nucleotide at position -3 of the acceptor dinucleotide AG, in a different fashion depending on the nature of this nucleotide (decreased binding to T resulting in exon exclusion, increased binding to C resulting in exon inclusion), whereas Q157 affects binding to the nucleotide at position +1 of the acceptor site (decreased with A, increased with G) [77,86,87]. Mutant U2AF1 induces missplicing of genes involved in cell cycle progression, RNA processing and of tumor-associated genes playing a significant role in myeloid leukemogenesis [86]. In MDS and AML, mutant U2AF1 favors the inclusion of exon 4 in *IRAK4*, a serine/threonine kinase mediating TLR signaling and NF-κB and MAPK activation [88]. This exon 4-containing isoform of *IRAK4* is more potent in activating NF-κB, by favoring MYDDosome assembly, thus playing a role in inflammation and leukemogenesis [88]. Also, a shorter variant of *PTBP1*, an alternative splicing regulator which interacts with pyrimidine-rich RNA sequences, is produced by *U2AF1* mutated cells [86]. This shorter variant of PTBP1 lacking a RRM domain, further affects global splicing regulation [86]. *U2AF1*^S34F^ knock-in models also display MDS features such as leukopenia and macrocytic anemia, but also myeloproliferative characteristics such as increased in vitro colony formation capacity [89,90].

*SF3B1,* also commonly mutated in MPN, especially those displaying ring sideroblasts, encodes a major component of the U2 snRNP [70]. Its role is to recognize the 3′-splice site at the intron junction [70]. *SF3B1* mutations mostly lead to amino-acid substitutions at restricted sites in H4-H8 repeats of the HEAT domain, which is involved in branch point recognition, the most frequent being K700E [70,76]. They favor the use of upstream cryptic branch points and cryptic 3′-splice sites, resulting in overall aberrant splicing [91,92]. Approximately half of aberrantly spliced mRNAs caused by *SF3B1* mutations result in down-regulation of gene expression, due to transcript degradation by NMD, while the other half would lead to the formation of neoproteins [91]. The affected transcripts vary depending on the pathology [93] and on the cell type. In MPN, *SF3B1* mutations give rise to splicing alterations in a specific set of transcripts, none of which has been clearly implicated in MPN pathogenesis. Interestingly, two genes (*OXA1L* and *SLC3A2*) display abnormal splicing after *SF3B1* mutation across 7 different types of cancer [93]. In MDS, core mitochondrial pathways are particularly affected by *SF3B1* mutations, notably because genes involved in mitochondrial ribosome and electron transport chain are often the targets of splicing anomalies leading to NMD [70]. Abnormal splicing of BRD9 is of particular interest. BRD9 is a component of the non-canonical BAF chromatin-remodeling complex [94] playing important roles in gene expression regulation [94,95]. An aberrant intronic branch point within *BRD9* intron 14 is recognized by mutated SF3B1 [94]. Whereas the alternative 3′ splice site in *SF3B1* mutated cells is generally located few bases upstream of the canonical site, resulting in the inclusion of a relatively short intronic sequence 5′ of the exon [91], the alternative acceptor site in *BRD9* intron 14 is further upstream, resulting in the inclusion of a whole alternative exon containing a STOP codon [94]. This “neo-exon” inclusion targets the transcript for degradation by NMD, thus decreasing BRD9 expression [94]. Decreased BRD9 expression impacts on ncBAF localization, thus altering gene expression regulation and DNA architecture [94]. *SF3B1*^K700E^ knock-in models recapitulate some aspects of MDS: macrocytic anemia, block in erythroid differentiation, increased LT-HSCs, but not the specific feature of this mutation in human diseases which are ring sideroblasts [96,97].

*ZRSR2*, located on the X chromosome, encodes a protein involved in the recognition of 3′ splice sites during the early stages of spliceosome assembly, especially of the minor spliceosome assembly [34]. Mutations of *ZRSR2* induce a loss of function and increase abnormal splicing characterized by U12-type intron retention (minor spliceosome) and few effects on U2-type introns [34]. Of particular interest are anomalies in the splicing of genes involved in MAPK signaling, erbB signaling, and in leukemogenesis, like *SRPK2*, *E2F*, *MAPK1*, *MAPK3*, *RAS* and *RAF*, altering proliferation and myeloid differentiation [34].

### 3.2. Splicing Anomalies in Myeloid Malignancies, Independent of Spliceosome Mutations

Beside MDS, splicing anomalies have been characterized in myeloid malignancies, including CML and AML. In CML, most of these anomalies seem to increase in accelerated and blastic phases, confirming a higher rate of missplicing in AML. Indeed, up to 30% of transcripts show splicing alterations when comparing CD34^+^ stem/progenitor cells from AML versus healthy donors [43]. Among the differentially spliced transcripts in AML, *FLT3*, a receptor tyrosine-kinase known to be frequently mutated in AML, displays frequent exon skipping between exons 6 and 8 leading to more potent activation of STAT5 and ERK [98]. Similarly, alteration of *NOTCH2* splicing (skipping of exons 12 or 17 and 18) observed in AML CD34^+^ cells, but not in normal control cells seems to alter its function, at least in terms of stimulation of *HES1* and *HEY1* transcription [98]. Even though the relation to leukemogenesis is unclear, it is interesting to note that these alternative splice variants provide prognostic information since patients with the splice form *NOTCH2-Va* have a poor outcome, especially in the intermediate risk cytogenetic group [98]. Overall, splicing anomalies seem to differ more broadly between AML patients with favorable versus unfavorable genetic characteristics, based on the study of large cohorts [44]. The splicing anomalies often affect genes involved in splicing themselves as well as protein translation [44]. These characteristics favor inflammation related genes, which could then account for prognostic differences [44]. Interestingly, comparing “young” versus “aged” human hematopoietic stem cells, Crews et al. [99] note that aging correlates with changes in alternative splicing favoring splicing deregulation and inflammatory responses (e.g., *CXCL2*), as well as epigenetic factors leading to myeloid skewing of the stem cells. Moreover, comparison of transcript expression with HSC from secondary AML patients shows further deregulation of splicing factors, inflammatory and anti-viral response pathways. This was true even in the absence of mutations in spliceosome genes [99]. Alternatively, spliced transcripts between aging and MDS/AML stem cells include genes already shown to be differentially spliced in blast crisis (BC) versus chronic phase (CP) of CML: *BCL-X*, *PTK2B*, *ITGB2*, *CD44* or *PTPN6*. Overall, splicing profiles allow discriminating young, aging and AML HSC [99].

The accumulation of splicing anomalies has also been reported in CML where BC cells display differentially spliced transcripts when compared to CP. This is well exemplified for *CD44*, an adherence molecule, the splicing of which varies depending on the maturation stage [40]. Progenitors from BC CML are enriched in the isoform specific to embryonic stem cells (*CD44v3*), thus showing that reprogramming of these progenitors to acquire self-renewal properties associate with a splicing program reminiscent of HSC [40]. Similarly, missplicing of *GSK3B*, involved in βCatenin-Wnt self-renewal signaling pathway, plays a role in the acquisition of stem cell properties in BC CML progenitors [100]. The transcription factor *GFI1B* (involved in megakaryocytic and erythroid differentiation) shows a transcript deprived of exon 9 in AML and CML, but not in normal or ET-derived PBMC [101]. It is unclear at present whether the protein encoded by this alternative transcript, displaying only four of the six zinc finger domains, and still active at promoting transcription in luciferase assays [101], plays a role in leukemogenesis. In contrast, the *BCL2L1* gene shows differential splicing in both AML and CML [99,102], promoting the anti-apoptotic form BCL-XL, thus explaining a survival advantage and TKI resistance [102]. Interestingly, the reversal of the switch toward *BCL-XL* overexpression using morpholino antisense oligomers restores apoptosis in response to TKI treatment, thus demonstrating that splicing anomalies can be targeted to increase treatment efficiency [103]. Last, a “full length” form of PYK2 (*PTK2B*), including alternative exon 23, is enriched in BCR-ABL1 expressing CD34^+^ cells (by transduction as well as in CP CML primary cells). This splicing event is controlled by BCR-ABL1 kinase activity and decreased by imatinib treatment. Although no experimental data confirms a functional difference, it is postulated that “FL PYK2” could be more potent than its naturally occurring counterpart devoid of exon 23 [104].

In Philadelphia-negative MPN, few studies report on splicing mechanisms, but some describe differential splicing of critical genes. A splicing isoform of *JAK2* itself, lacking exon 14 where is located the V617F mutation, has been reported in MPN patients [105,106]. In 2010, Ma et al. described this isoform exclusively in plasma of MPN patients, but not of healthy controls [105]. Fifteen percent of the MPN patient cohort (*n* = 61) displayed significant amounts of this short isoform detected by fragment analysis, which represented 4–34% of total *JAK2* transcripts [105]. Catarsi et al. [106] also detected this isoform in granulocyte RNA, but it was also observed in normal healthy controls. However, they reported a higher proportion of transcripts missing exon 14 in *JAK2*^V617F^-mutated MPN whereas this proportion was identical in *JAK2*^V617F^-negative MPN and in healthy controls [106]. Moreover they describe a correlation between *JAK2*^V617F^ allelic burden and the proportion of the short isoform [106]. In our hands, the alternative isoform of JAK2 was also found in MPN patients at a higher level than in healthy controls or patients with reactive thrombocytosis, the highest levels being observed in PMF patients as opposed to other MPN (our unpublished results). In silico analyses predicted that *JAK2*^V617F^ mutation led to a switch in the binding affinity between SRSF2 and SRSF6 that may explain exon skipping [107]. However, in our hands, modification of SRSF2 expression did not alter the skipping of exon 14 on a minigene model, but SRSF6 overexpression did, whether the V617F mutation was present or not (our unpublished results).

Another example of splicing modification affects death inducer-obliterator (*DIDO*). This transcription factor gene can give rise to three alternative transcripts: *DIDO1*, *DIDO2* and *DIDO3* [108,109,110]. DIDO1 favors apoptosis by upregulation of procaspase 3 and 9 [111], while DIDO2 and DIDO3 do not. DIDO3, the major isoform, is involved in genomic stabilization, in preventing supernumerary centrosomes, maintaining cellular mitotic arrest and functional mitotic checkpoint [109]. In embryonic stem cells, the relative proportion of DIDO1 and three have been reported to modulate the self-renewal/differentiation balance [112]. Patients with MDS/MPN or MPN showed decreased expression of all *DIDO* transcripts, which do not affect similarly *DIDO1*, *2* or *3* [108]. Of note, *DIDO* knock-down in mice results in a phenotype reminiscent of MDS/MPN [108]. It is unclear however, whether global decrease of DIDO expression or specific variation of one of its isoforms is involved in this phenotype.

MPL, the receptor for thrombopoietin (TPO) plays a major role in MPN pathogenesis. It has multiple splicing isoforms due to inclusion or exclusion of exons 8 and 9 (*MPL-D*) [113], 9 and 10 (*MPL-Tr*) [114,115] or intron 10 retention (*MPL-K*) [115]. MPL-K is devoid of intracellular domain [115,116], but does not have dominant negative effect [117] possibly due to its inability to dimerize with full length (FL) MPL. In contrast, MPL-Tr, devoid of the WSXWS and part of the transmembrane domain [114,115], does show dominant negative effect, probably by increasing MPL degradation by the lysosomal pathway [118]. Last, MPL-D, the extracellular domain of which is affected, has increased affinity for TPO [119], but low expression at the cell surface [113], probably due to its degradation after TPO binding and internalization. However, this form is able to stimulate ERK1/2 more potently than FL MPL [119]. To date, it is not clear whether abnormal distribution of these different splice isoforms contribute to MPN phenotype or are modified upon disease evolution.

## 4. Mechanisms Driving Splicing Anomalies in the Evolution of MPN

We have just seen that altered splicing can have consequences participating in leukemogenesis. We have also seen that in CML and AML, most deregulated splicing events are independent of spliceosome mutations. We can then ask what mechanisms are responsible for these splicing anomalies, especially with the aim of reversing these effects for a potential therapeutic interest. Pre-mRNA splicing patterns differ between cells depending, between others, on their maturation stage [120], and even for a given cell population, depending on the age of the patient [99]. Many factors are susceptible of modifying these splice patterns in cancers, including stress, as has been described following *MYC* overexpression [121]. Since *MYC* overexpression has been shown to participate in MPN pathogenesis, and especially in blastic phases, MYC- or other stressors-induced splicing changes could have relevance in MPN transformation [122,123].

The molecular mechanisms explaining these splice pattern changes in cancers are varied, but seem to mostly rely on epigenetic modifications and/or dysregulation of splice regulators. Epigenetic modifications include DNA methylation, which can occur at promoters or in the body of the genes. Differential DNA methylation directs recruitment of proteins involved in exon determination (such as MeCP2 or HP1), thus playing a role in exon inclusion or exclusion [124]. Moreover, DNA methylation, by orchestrating chromatin architecture (nucleosome recruitment), plays a major role in determining transcription rate. It has been particularly well established that transcription rate is crucial for the “choice” of exon inclusion/exclusion [41]. Interestingly, in MPN, it has been reported that JAK2 was able to phosphorylate Y41 of histone 3, thus preventing HP1 binding and altering chromatin structure [125]. This could be a way by which MPN driver mutations could affect splicing. It is probable that epigenetic related splicing modifications are even more important after mutation of epigenetic regulators. For instance, *TET2* or *IDH1/2* mutations lead to a change in 5-hydroxymethyl-cytosine content. Hydroxymethylation in exons has been shown to favor exon inclusion in granulocytes [126].

Epigenetic regulation of gene expression and splicing relies on DNA methylation/hydroxymethylation, but also on histone post-translational modifications, which are, to some extent, interrelated. For instance, comparing young and aging HSC, Sun et al. [127] showed that aging is associated with increased H3K27 methylation and CpG methylation of polycomb targeted genes and these marks are associated with the suppression of a short isoform of Nr4a2, a regulator of HSC quiescence [127]. Post-translational histone modifications are also believed to be the basis of *MPL-Tr* specific splicing: the RNA binding protein RBM15 can associate with Histone Deacetylase 3 and/or Histone Methyltransferase SetD1b and with *MPL* pre-mRNA to favor *MPL-Tr* isoform [128].

Epigenetic mutations such as those affecting *IDH1/2* modify DNA hydroxymethylation and histone codes. Interestingly, *IDH1/2* mutations have been shown to increase splicing anomalies, not only when they occur simultaneously with *SRSF2* mutations (then increasing splicing anomalies) [129], but also in a murine model in which *JAK2* and *IDH2* mutations are combined (our analysis of RNA-seq data presented in [130]). Interestingly, as was the case with spliceosome mutations, the main pathways affected by IDH-deregulated splicing involve genes playing themselves a role in splicing, thus generating a global splicing deregulation.

Last, RNA-editing mechanisms may lead to a change in an important base for exon recognition. This mechanism explains the abnormal splicing of *GSK3B* in CML cells: during CML progression, IFNγ- and BCR-ABL1-stimulation of expression of ADAR1 results in a propensity to change A to I nucleotides, possibly affecting splice donor or acceptor sites [131].

In addition to the regulation by epigenetic mechanisms, splicing is controlled by many regulator proteins/ribonucleoproteins. The expression of these factors can change depending on cell type and maturation stage and/or in response to oncogenic stressors. An example is provided by the decreased expression of the RNA-binding protein MBNL3 in leukemic stem cells (LSC) isolated from blast crises of CML. MBNL3 is implicated in the repression of a splicing pattern specific to embryonic stem cells and its reduced expression is associated with reappearance of this embryonic pattern as demonstrated by the expression of the v3 isoform of CD44 [40]. Other proteins involved in splicing regulation probably play a role in MPN deregulated splicing. For instance, it is of interest that SR proteins, major regulators of exon inclusion, can be modulated by post-translational modifications, including phosphorylation by PI-3 kinase, a pathway stimulated by MPN driver mutations [132]. Also, protein arginine methyl transferases (PRMT) methylate various substrates, including spliceosome components. It has been recently shown that PRMT5 modulation, including after stimulation of interferon receptors, modifies splicing programs in lymphocytes [133]. In MPN, the mutant JAK2^V617F^ protein, but not wild type JAK2, can phosphorylate PRMT5, thus reducing its methyl-transferase activity [134]. In addition to the well characterized effects of PRMT5 on epigenetic regulation (histone methylation), it is likely that JAK2^V617F^-mediated PRMT5 phosphorylation also alters splicing programs. This research domain is of particular interest since PRMT inhibitors are the subject of active therapeutic research in cancers.

## 5. Therapeutic Targeting of Splicing Anomalies

The importance of splicing anomalies in the pathogenesis of MPN has stimulated the research for specific therapeutic approaches, targeting the splice sites, the spliceosome itself or splicing regulators. Modified oligonucleotides have been used to mask specific splice sites, thus favouring alternative sites. For instance, in CML and AML, *BCL2L1* splicing favours the anti-apoptotic protein BCL-XL, responsible for resistance to TKI. This is due to inclusion of an alternative exon 2b, the 3′ splice site of which can be targeted by a morpholino-modified oligonucleotide, thus preventing its recognition by the spliceosome [103]. As a result, the BCL-XS form, devoid of exon 2b is preferentially expressed. Using this strategy, Zhang et al. [103] have documented a synergic effect with imatinib for induction of apoptosis in K562 cells and suppression of subcutaneous tumours in K562 xenograft models. Overall, trials with these “splice-switching oligonucleotides” (SSO) seem more promising in constitutional monogenic diseases than in cancers where many splicing events cooperate. However, attempts at redirecting splicing of genes involved in splicing regulation or their target sequences (such as SRSF2), thus broadening the targets, may be of interest [135].

Another theoretical possibility of targeting sequences in *cis* to modulate splicing is the pharmacological modulation of RNA editing enzymes, such as ADAR which has been shown to play a role in *GSK3B* missplicing in CML [131]. To our knowledge, no such development has been made to treat myeloid malignancies.

In order to reach several targets, pharmacological modulation of the spliceosome is a valuable strategy. PRMT5 participates in the assembly of early spliceosome through arginine-methylation of ribonucleoproteins (mainly Sm proteins) [136]. This symmetric arginine methyltransferase has many other effects on cell biology, including transcriptional regulation via histone methylation and it impacts on chromatin remodeling and transcriptional repressor complexes [136]. PRMT5 has been shown to be overexpressed in CML LSC, and its inhibition to alter stem cell functions such as serial replating. Consequently, in mouse models of CML (by transplantation of *BCR-ABL1*-transduced bone marrow cells) PRMT5 knock-down or pharmacological inhibition increases mice survival [137]. This effect seems to rely on decreased p15 and p27 tumor suppressor expression through recruitment of DNMT3A [137]. It is unclear whether splicing regulation plays a role in this model.

More interestingly, PRMT5 inhibitors have been shown to decrease MPN burden in mice transplanted with hematopoietic stem cells expressing *JAK2*^V617F^ or *MPL*^W515L^ [138]. In this study, the effect seems to mainly rely on E2F1 transcription factor dimethylation, which impairs its binding to RB, thus deregulating the cell cycle [138]. However, the importance of splicing modulation after PRMT5 inhibition is suggested by the fact that PRMT5 inhibitors kill more efficiently SRSF2^P95H^-mutated cells than SRSF2^WT^ control cells in a model of acute leukemia due to *MLL-AF9* gene fusion [139]. In addition, PRMT5 inhibitors synergize with PRMT1 inhibitors or other spliceosome inhibitors such as E7107 in this apoptosis induction which then extends to *SRSF2*^WT^ *MLL-AF9*-positive cells [139]. Interestingly, in this study, RNA-seq analyses show that combined inhibition of PRMT5 and PRMT1 reverse the *EZH2* poison exon inclusion characteristic of SRSF2^P95H^ mutated cells.

PRMT1 has also been implicated in splicing regulation by favouring ubiquitination of RBM15, thus increasing the short *RUNX1a* transcript, a dominant negative form of this transcription factor [140]. Its inhibition seems interesting in acute leukemias since PRMT1 increases the transcriptional activity and self-renewal of cells with *KMT2A* (*MLL*) or *RUNX1-RUNX1T1* (*AML1-ETO*) rearrangements [141]. It can also activate the tyrosine-kinase activity of FLT3 in primary acute lymphoblastic leukemia cells [142]. These properties make it an interesting target for cancer treatment, possibly in association with PRMT5 inhibitors, as mentioned above. It is unclear whether PRMT1 inhibitors effects in cancers is due to their functions on splicing, but it is interesting to note that SRSF9 can be methylated by PRMT1, thus affecting its cellular sublocalization [143].

Several families of molecules, initially derived from bacteria, are more specific inhibitors of the spliceosome function. These include pladienolides (with E7107 and H3B-8800), herboxidienes and spliceostatins (with sudemycins), which all interact with the SF3B complex [135]. Pre-clinical and clinical results with these drugs have been extensively reviewed [135,144,145]. The E7107 molecule seemed promising since in mouse models of AML bearing a mutated *SRSF2* gene (inducible knock-in or patient cell xenografts), this spliceosome inhibitor killed more efficiently SRSF2-mutated cells. The same was true with ZRSR2 mutants. The main splicing anomalies following this treatment were intron retention and cassette exon exclusion, which were more pronounced in spliceosome mutated cells than in controls [146]. Unfortunately, the first clinical trials with this molecule have been complicated with vision loss in several patients, thus hindering its development [145]. The H3B-8800 derivative seems to have similar effects on splicing with increased intron retention, mainly affecting short, GC-rich introns, especially in genes involved in RNA metabolism [147]. This molecule is currently in early phases of clinical trial in MDS/AML. In addition to globally disrupting splicing, which is presumed to be lethal in cells harbouring a spliceosome mutation, these SF3B inhibitors may have specific effects in cells that have developed resistance to kinase inhibitors. For instance, melanoma cells carrying a *BRAF*^V600E^ mutation may become resistant to vemurafenib by favouring an alternative transcript lacking exons 4–8 (encoding a Ras-binding domain). This alternative isoform is due to a point mutation near the branch point of intron 8. Interestingly, treatment of these cells with spliceostatin A or its derivative meayamycin B resulted in restoration of normal *BRAF* splicing and BRAF inhibitor sensitivity [148].

Splicing regulation mainly relies on RNA binding proteins which interact with splicing inhibitors/enhancers, among which SR protein family members. These factors can be post-translationally modified, which affects their function. These post-translational modifications can be pharmacologically targeted. Several compounds, identified by in vitro screens as inhibitors of SR protein kinases (SRPK) or Cdc2-like kinase (CLK), both involved in SR protein phosphorylation, may have some therapeutic potential [135]. Other post-translational modifications impact on splicing regulation, including ubiquitination. SRSF6 ubiquitination is deficient in T-ALL, resulting in altered exon inclusion, mainly affecting proteins with proteasomal function, or involved in RNA metabolism or cell cycle regulation. Spliceosome inhibitor (H3B-8800) showed synergistic effects with proteasome inhibitors in killing T-ALL cells [149].

Beside SR proteins, other splicing regulators might be the targets of specific therapies. For instance, the RNA binding protein MBNL1 is overexpressed in *KMT2A (MLL)* rearranged leukemias. This results in splicing anomalies, mainly intron retention (a hallmark of cancer splicing programs) that is reversed upon MBNL1 downregulation, which might be a future therapeutic target [150].

Last, RBM39, a ribonucleoprotein involved in splicing through interaction with U2AF2 and SF3B1, as well as a regulator of SRSF10 and hnRNPH1, has been shown to be overexpressed in AML cells as compared to normal CD34^+^ cells. Its down-expression impairs correct splicing (as assessed by RNA-seq) and cell survival. It is thus of therapeutic interest to decrease its expression. Interestingly, indisulam, a small molecule identified by a screen of potential anti-cancer molecules, was recently shown to stabilise a DCAF15 complex which acts as a ubiquitin ligase for RBM39 [151]. Thus, indisulam favours RBM39 ubiquitination and proteasomal degradation. This results in a major cytotoxicity in AML cells, but not in normal progenitors and a reduction of leukemic burden and improved survival in mice engrafted with AML cells. Interestingly, AML cell lines in which mutations of *SRSF2*, *SF3B1* or *U2AF1* have been introduced are more sensitive to indisulam-induced cell death than their non-mutated counterparts [152].

## 6. Conclusions

In conclusion, pre-mRNA splicing is a complex highly regulated phenomenon, providing a large variety of transcripts, the distribution of which is specific to each cell type and maturation stage. Abnormal splicing is a hallmark of cancer including myeloid malignancies. Well studied in MDS, mainly after the discovery of recurrent spliceosome mutations, it is not very well characterized in MPN. However, the accumulation of splicing anomalies during CML evolution as well as the demonstration of abnormal splicing in AML suggests that splicing deregulation is also of importance in Philadelphia-negative MPN, especially in advanced phases. The clinical efficacy of splicing inhibitors in spliceosome mutated MDS or AML demonstrate that therapeutic targeting of splicing could open new venues for treatment of MPN advanced phases, for which current therapeutic tools are unsatisfactory. Similarly, the proof of concept of the efficacy of oligonucleotides to re-direct “pro-leukemogenic” splicing anomalies could also pave the way for future strategies. A better understanding of mechanisms at play in splicing deregulation in MPN is necessary to design new therapeutic tools and possibly select “splicing biomarkers” predictive of pejorative evolution.

## Figures and Tables

**Table 1:** Spliceosomal mutations in MPN and AML post-MPN patients.

Gene	Mutation Frequency (n/N)					References
	PMF	SMF	ET	PV	AML Post MPN	
*SRSF2*	12.2% (351/2876) *Pre-MF*: 8.5% (25/294)	2.6% (12/461)	1.5% (31/2132)	1.8% (22/1244)	17% (25/147)	[24,46,47,48,49,50,51,52,53,54,55,56,57,58,59,60,61]
*SF3B1*	7.7% (135/1751)	6.9% (8/116)	2.8% (62/2207)	1.4% (14/1027)	8.1% (13/161)	[45,46,47,48,49,50,51,53,55,56,57,58,59,60,61,62,63,64]
*U2AF1*	13.8% (200/1448)	4.9% (5/102)	1.1% (23/2132)	0.3% (3/969)	9.5% (14/147)	[46,47,48,49,50,53,55,56,57,58,59,60,61]
*ZRSR2*	5.8% (52/891)	6.9% (7/102)	0.7% (11/1590)	1.3% (7/535)	1.1% (1/91)	[46,47,48,55,56,57,58,59,61]
*PRPF8*	2.1% (2/94)	1.6% (1/64)	2.9% (1/35)	0% (0/8)	5.3% (5/94)	[48,50,59]

## Supplementary Materials

The following are available online at https://www.mdpi.com/2072-6694/12/8/2216/s1, Table S1: Co-occurrences of mutations in PV, ET, MF and post-MPN AML.
